# Chemical Investigation of *Crataeva nurvala* Buch. Ham. Fruits

**DOI:** 10.4103/0250-474X.54276

**Published:** 2009

**Authors:** Gagandeep, Meera, S. B. Kalidhar

**Affiliations:** Department of Chemistry, Haryana Agricultural University, Hisar-125 004, India

**Keywords:** *Crataeva nurvala*, chemical components, pentadecane, octanamide, 12-tricosanone, friedelin

## Abstract

Chemical investigation of fruits of *Crataeva nurvala* has revealed the presence of four known compounds which are pentadecane, octanamide, 12-tricosanone and friedelin. These compounds have been characterized on the basis of spectral and other data. These are being reported for the first time from the fruits of this plant.

*Crataeva nurvala* (family: Capparidaceae) is commonly known as *barna* and *varuna*[1]. It is distributed, wild or cultivated, throughout India and tropical regions of the world[2]. Fruits are berry-like, globose or oblong; edible and used as astringent[3]. Fruiting occurs in April-June. Seeds are embedded in yellow pulp. Rind of the fruit is used as a mordant in dyeing[4]. As the fruits of the plant have not been extensively studied for chemical components, the present study was taken up.

Melting points were determined on a Ganson Electrical Melting Point Apparatus. ^1^H NMR spectra were recorded on a Bruker AC 300 MHz NMR Spectrometer using TMS as an internal standard. Chemical shifts are given in δ (ppm) and CDCl_3_ was used as solvent. IR spectra were recorded on a Hitachi 570 Infrared Spectrophotometer. Mass Spectra were obtained on a VG 70 S 11-250 J GCMS-DS Spectrometer.

Fruits of *C. nurvala* (5 kg) were collected from Botanical Gardens, HAU, Hisar. These were crushed, dried and extracted with hot methanol. The plant material (1 kg at a time) had been taken into a 5 l RB flask fitted with a water condenser for refluxing the material with MeOH. Refluxing was carried out for 6 h. The process had been repeated three times to prepare methanol extract of 5 kg of the material. The methanol extract was concentrated over water bath under reduced pressure. Extractives were subjected to silica gel (60-120 mesh) column chromatography. Four compounds were obtained.

Compound A (n-pentadecane, 1), molecular formula C_15_H_32_, was obtained on elution with petroleum ether as an oily liquid, 5 ml, b.p. 270°[5]. IR (KBr, v_max_, cm^−1^): 600, 800, 1026, 1261, 1378, 1441, 2362. ^1^H NMR (CDCl_3_, δ): 1.25 (26 H, br, 13* -CH_2_-), 0.88 (6 H, t, *J* 7.5 Hz, 2* -CH_3_). GCMS (m/z): 212 (M^+^).

Compound B (octanamide, 2), molecular formula C_8_H_17_NO, was obtained on elution with benzene-petroleum ether (1:3) as a colourless solid, 10 mg, m.p. 112° (literature m.p. 110-112°)[6]. IR (KBr, v_max_, cm^−1^): 756, 802, 1026, 1235, 1372, 1740, 2925, 3456. ^1^H NMR (CDCl_3_, δ): 5.13 (2 H, s, CONH_2_), 2.02 (2 H, t, *J* 7.5 Hz, -C*H_2_*CONH_2_), 1.66 (2 H, br, -*CH_2_*CH_2_CONH_2_), 1.25 (8 H, br, 4* -CH_2_-), 0.88 (3 H, t, *J* 7.5 Hz, 2* -CH_3_). GCMS (m/z): 143 (M^+^).

Compound C (12*-tricosanone*, 3), molecular formula C_23_H_46_O, was obtained on elution with benzene as a colourless solid, 10 mg, m.p. 69° (reported m.p. 68°)[6]. IR (KBr, v_max_, cm^−1^): 765, 1012, 1257, 1460, 1617, 1733, 2916. ^1^H NMR (CDCl_3_, δ): 2.33 (4 H, *J* 7 Hz, -CH_2_CO-), 1.54 (4H, m, 2* -C*H_2_*CH_2_CO-), 1.26 (32 H, br, 16* -CH_2_-), 0.88 (6 H, t, *J* 7.0 H_z_, 2* -CH_3_). GCMS (m/z): 338 (M^+^).

Compound D (friedelin, 4), molecular formula 426, was obtained on elution with ethyl acetate-benzene (1:19) as a colourless crystalline solid, 15 mg, m.p. 260° (reported m.p. 259-261°)[7]. It gave pale brown colour on reaction with Ac_2_O/H_2_SO_4_. IR (KBr, v_max_, cm^−1^): 980, 1053, 1073, 1176, 1205, 1220, 1377, 1389, 1715. ^1^H NMR (CDCl_3_, δ): 1.25-2.31 (25 H, m, 11*-CH_2_-, 3* -CH<), 1.16 (3H, s, -CH_3_), 1.05 (3H, s, -CH_3_), 1.01 (6 H, 2* -CH_3_), 0.97 (3H, s, -CH_3_), 0.88 (6H, s, 2* -CH_3_). GCMS (m/z): 426 (M^+^).

The four known compounds are being reported for the first time from the fruits of this plant.
